# Ozone therapy promotes the differentiation of basal keratinocytes via increasing Tp63‐mediated transcription of KRT10 to improve psoriasis

**DOI:** 10.1111/jcmm.15160

**Published:** 2020-03-13

**Authors:** Lihua Gao, Jianhua Dou, Bo Zhang, Jinrong Zeng, Qingmei Cheng, Li Lei, Lina Tan, Qinghai Zeng, Shu Ding, Aiyuan Guo, Haipeng Cheng, Caifeng Yang, Ziqiang Luo, Jianyun Lu

**Affiliations:** ^1^ Department of Physiology College of Basic Medicine Central South University Changsha China; ^2^ Department of Dermatology The Third Xiangya Hospital Central South University Changsha China; ^3^ Department of Stomatology The Third Xiangya Hospital Central South University Changsha China

**Keywords:** keratin 6 (KRT6)/KRT10, keratinocytes (KCs), ozone therapy, psoriasis, Tp63

## Abstract

Psoriasis is a chronic immune‐mediated inflammatory dermatosis. Recently, ozone therapy has been applicated to psoriasis treatment; however, the mechanism by which ozone therapy improves psoriasis remains unclear. The excessive proliferation and the differentiation of basal keratinocytes have been considered critical issues during pathological psoriasis process, in which keratin 6 (KRT6) and KRT10 might be involved. In the present study, KRT6, IL‐17 and IL‐22 protein within psoriasis lesions was decreased, while KRT10 and Tp63 protein in psoriasis lesions was increased by ozone treatment in both patient and IMQ mice psoriatic tissues. In the meantime, ozone treatment down‐regulated KRT6 mRNA and protein expression while up‐regulated KRT10 mRNA and protein expression within IL‐22 treated primary KCs; the cell viability of KCs was suppressed by ozone treatment. Moreover, Tp63 bound to KRT10 promoter region to activate its transcription in basal keratinocytes; the promotive effects of ozone on Tp63 and KRT10 were significantly reversed by Tp63 silence. Both TP63 and KRT10 mRNA expression were significantly increased by ozone treatment in psoriasis lesions; there was a positive correlation between Tp63 and KRT10 expression within tissue samples, suggesting that ozone induces the expression of Tp63 to enhance the expression of KRT10 and the differentiation of keratinocytes, therefore improving the psoriasis. In conclusion, the application of ozonated oil could be an efficient and safe treatment for psoriasis; ozone promotes the differentiation of keratinocytes via increasing Tp63‐mediated transcription of KRT10, therefore improving psoriasis.

## 1 INTRODUCTION

Psoriasis is a common recrudescent chronic immune‐mediated inflammatory dermatosis, which is characterized by hyperplasia of epidermis, angiogenesis, and soakage of inflammation cells.^1^ Psoriasis can lead to physical and psychological trauma to patients, therefore affecting up to 2%‐4% of the global population.^2^, ^3^, ^4^ A number of treatments that have been used to treat psoriasis include topical preparations containing corticosteroids, retinoid derivatives, synthetic vitamin D3 analogs, tar, or anthralin; systemic drugs, including immunosuppressants and calcineurin inhibitors cyclosporine, tacrolimus, acitretin and isotretinoin; as well as photochemotherapy (PUVA) and UVB irradiation.^5^, ^6^, ^7^ However, most psoriasis therapies bring up considerable side effects, including skin atrophy, telangiectasia, purpura, hairy and folliculitis.^8^, ^9^, ^10^

Ozone (O_3_) is a strong oxidizing molecule composed of three oxygen atoms. Ozone has anti‐infection, accelerates blood metabolism, anti‐inflammatory^11^ and immunomodulatory effects,^12^, ^13^ and can be used in a wide range of diseases. Recently, the ozone therapy has also been applied to psoriasis treatment.^14^, ^15^ Tan et al^15^ prepared ozonated oil by dissolving ozone in vegetable oil and compared the efficacy and safety between ozonated oil and compound flumethasone ointment in the treatment of psoriasis vulgaris. After 4‐week treatment, the epidermis was approximately normal and few inflammatory cells infiltration in the dermal papilla were observed in both groups. They concluded that the ozonated oil treatment for stable psoriasis is safe and effective, and its efficacy is equivalent to the effect of glucocorticoid topical preparations. Regarding the molecular mechanism, another group has speculated that O_3_ could affect the activity of MMP,^14^, ^16^, ^17^ thus exerting the effects not only on the occurrence and development of disorders associated with human skin ageing,^18^, ^19^, ^20^ but also on other skin diseases including psoriasis and dermatitis.^14^, ^15^, ^21^, ^22^, ^23^, ^24^, ^25^ However, the precise mechanism by which the ozone therapy improves psoriasis remains unclear.

The basal keratinocytes in the lesions of psoriasis proliferate rapidly, and they do not fully differentiate into the epidermal horny layer to form hyperplasia, while the undifferentiated basal keratinocytes in normal skin undergo a long period of process such as proliferation, differentiation and evolution, and eventually enter the cuticle of the epidermis. Studies have found that ozone may affect basal keratinocyte differentiation. The expression of KRT10 (KRT10) is increased after ozone treatment of skin tissue, while KRT10 is a keratin marker produced in well‐differentiated upper basal keratinocytes.^13^, ^26^ In addition, KRT10 and other differentiation markers are one of the characteristics of psoriasis.^27^ KRT10 expression could be significantly down‐regulated within lesions and marginal healthy skin cells of diffuse psoriasis; more keratin 6 (KRT6)‐positive and KRT10‐negative cells were detected in the inner margin of the lesions.^28^ Therefore, we speculate that ozone may directly affect the pathological process of psoriasis through the regulation of the differentiation of basal keratinocytes and that KRT6/10 might be involved.

Regarding the underlying mechanism of KRT10 deregulation in psoriasis lesions, Tp63 was predicted to bind KRT10 promoter region in keratinocytes via the Chip‐Atlas (http://chip-atlas.org/) in the chromatin co‐immunoprecipitation database (http://ddbj.nig.ac.jp/). Reportedly, Tp63 could regulate the ability of keratinocytes to proliferate and differentiate.^29^, ^30^ Full‐length Tp63 could be remarkably inhibited within lesional tissue than that in clinically normal skin from patients and matched healthy controls.^31^, ^32^ More important, Tp63 is involved in the regulation of redox status and is regulated by oxidative stress in epithelial cell, including in keratinocytes.^33^ Therefore, we speculate that ozone induces Tp63 activation, promotes KRT10 expression and accelerates basal keratinocyte differentiation, therefore improving the psoriatic lesions.

Herein, the efficiency of ozone in the treatment of psoriasis has been further proved. The effects of ozone on the differentiation of basal keratinocytes, as well as the protein levels of KRT6 and KRT10, were also examined. Primary basal keratinocytes, KCs, were then stimulated with IL‐22 to establish a cell model of psoriasis^34^, ^35^ and treated with ozone and examined for cellular morphological changes and the protein levels of KRT6 and KRT10. Next, the predicted binding between Tp63 and KRT10 promoter region was validated by ChIP and luciferase reporter assays. The effects of Tp63 silence on Tp63 and KRT10 proteins were examined under ozone treatment. Finally, the mRNA expression of Tp63 and KRT10 was examined in psoriasis lesion tissues with or without ozone treatment; we examined the interaction between the expression of Tp63 and KRT10 within tissues using Pearson's correlation analysis. In summary, we provide experimental basis for a novel mechanism by which the ozone therapy improves the psoriasis via regulating the differentiation of basal keratinocyte.

## 2 MATERIALS AND METHODS

### 2.1 Tissue samples

Twenty psoriasis vulgaris patients (age 38.25 ± 4.31, male/female: 16/4) diagnosed by dermatologists according to diagnostic standard were recruited to participate in this study from April 2016 to January 2019 with the approval of the Research Ethics Committee of the Third Xiangya Hospital. Patients’ lesion skin was smeared with ozonated oil (containing 13.5% ozone, Cat: 20160317, 20161123, 20170728, 20180515, Hunan Haizhi Medical Scientific Company) twice a day for 4 weeks. Twenty lesion skin tissues of patients treated or not treated with O_3_ were collected from the Third Xiangya Hospital. PASI scoring is based on 20 patients received ozone therapy. Informed consents were obtained. Tissue samples were fixed in formalin or stored a −80℃ until the further study.

### 2.2 Cell line and cell transfection

Primary healthy human KCs were obtained from ATCC (ATCC^®^ PCS‐200‐011™, ATCC). Cells were cultured in Medium 154 (M154500; Gibco) supplemented with Human Keratinocytes Growth Supplement (S0015, Gibco). All cells were maintained at 37°C in a humidified tissue culture incubator with 5% CO_2_. KCs were stimulated with 100 ng/mL human recombinant cytokines IL‐22 (13059‐HNAE; Sino Biology) as described previously^36^ to mimic the cellular alterations during psoriasis‐like dermatitis. For O_3_ treatments, ozone gas was dissolved in PBS solution to maintain a final concentration of 0.3 mg/L. Ozonated PBS was added to the medium, the cells were further cultured for 30 minutes, and the medium was changed. The above operations were performed once a day for a total of 3 days.

The silence of Tp63 was achieved by transfection of si‐Tp63 (Genepharma). The overexpression of TP63 was achieved by transfection of pcDNA3.1/TP63 (Genepharma) with the help of Lipofectamine 3000 (Invitrogen).

### 2.3 Psoriasis‐like dermatitis mouse model

Psoriasis‐like dermatitis model was established in healthy BALB/C mice. Mice were randomly divided into the blank, imiquimod group (IMQ), IMQ + vehicle group and IMQ + Ozone group. IMQ cream (62.5 mg, 5%) was applied following the methods described previously.^37^ For O_3_ therapy, lesion skin was smeared with ozonated oil  once a day for 1 week. The morphological changes were photographed and evaluated for the total sign score (TSS).^38^ The pathology changes were examined using H&E staining. The protein expression level of IL‐17, IL‐22, Tp63, KRT6 and KRT10 in skin tissues was examined by immunohistochemistry (IHC) staining.

### 2.4 Haematoxylineosin (H&E) staining

Samples were fixed in 4% paraformaldehyde for 24 hours, dehydrated, embedded in paraffin, and cut into 4‐μm‐thick slices. Slices were heated at 37°C overnight, dewaxed, and stained with H&E following the methods described previously.^39^, ^40^

### 2.5 IHC staining

To evaluate the protein contents of IL‐17, IL‐22, Tp63, KRT6 and KRT10, tissue sections were fixed in acetone for 10 minutes at −20°C, permeabilized with 0.2% triton (Sigma) for 10 minutes at room temperature, incubated with a blocking solution (3.75% BSA/5% goat serum, Zymed) for 30 minutes and incubated for 2 hours with IL‐17 (26163‐1‐AP; Proteintech), IL‐22(ab203211; Abcam), Tp63 (PA2056; Bosterbio), KRT6 (10590‐1‐AP; Proteintech) or KRT10 (sc‐53252; Santa Cruz). Control sections were incubated with only blocking solution or appropriate mouse or rabbit IgG isotype control antibodies. All sections were incubated with HRP‐secondary antibodies (Bosterbio) for 1 hour at room temperature. Diaminobenzidine (DAB) staining kit (Bosterbio) was applied to visualize the target proteins and haematoxylin staining was used to re‐stain the nucleus. The sections were observed by optical microscope.

### 2.6 Immunoblotting assays

Protein samples were separated by SDS‐PAGE, transferred from gel to a PVDF membrane. The membrane was incubated with antibodies against KRT6, KRT10 and Tp63 following the methods described previously.^41^ After incubating with the primary antibodies mentioned above, the blots were incubated for 1 hour with HRP‐conjugated goat anti‐mouse or goat anti‐rabbit IgG (Beyotime). Protein levels in each lane were normalized to the levels of GAPDH.

### 2.7 PCR‐based analysis

Total RNA was extracted from cultured cells using Trizol reagent (Invitrogen). The expression of mRNA was measured using a SYBR Green qPCR assay (Takara). The expression of GAPDH served as an endogenous control. The 2^−ΔCT^ method was applied for data processing.

### 2.8 Immunofluorescence

Treated target cells were plated on a glass slide at a density of 1 × 10^4^ cells/mL, fixed by 4% precooled formaldehyde, washed by PBS containing 0.1%Triton X‐100 for three times and then blocked with 5% BSA at room temperature for 2 hours. The slides were incubated with anti‐KRT6 or anti‐KRT10 antibodies for 1 hour at 37°C and with Cy5 or FITC labelled secondary antibodies (Beyotime) for 1 hour at room temperature. The nucleus was staining by DAPI (Beyotime) for 5 minutes at room temperature. The slides were observed by inverted fluorescence microscope (Olympus).

### 2.9 Chromatin immunoprecipitation (ChIP)

To validate the binding between Tp63 and KRT10 promoter region, ChIP assays were performed using anti‐Tp63 (Cat.687203, Biolegend) following the methods described previously.^42^ RNA polymerase II was used as a positive control antibody and non‐immune IgG was used as a negative control demonstrate the efficacy of the kit reagents (Epigentek Group Inc, P‐2025‐48). The immunoprecipitated DNA was eluted and examined for fold‐enrichment according to the methods described previously.^42^

### 2.10 Luciferase reporter assay

KRT10 promoter, harbouring the predicted TP63 binding site, was cloned to psiCheck‐2 (Promega) to construct KRT10 promoter luciferase reporter vector (psiCheck‐2‐proKRT10). Cells were co‐transfected with pcDNA3.1‐TP63 or blank pcDNA3.1 and psiCheck‐2‐proKRT10 luciferase reporter vector; after 24 hours, the activities of firefly luciferase and Renilla luciferase were measured in the cell lysates using a Dual‐Luciferase Assay System (Promega).

### 2.11 Statistical analysis

Results from at least three independent experiments were processed using GraphPad and then expressed as means ± SD. Data were statistically analysed by one‐way analysis of variance (ANOVA) followed by Tukey's multiple comparison test or independent sample *t* test. A *P*‐value of <.05 was considered as statistically different.

## 3 RESULTS

### 3.1 Clinical ozone (O_3_) therapy improves psoriatic lesions in vivo

**Figure 1:**
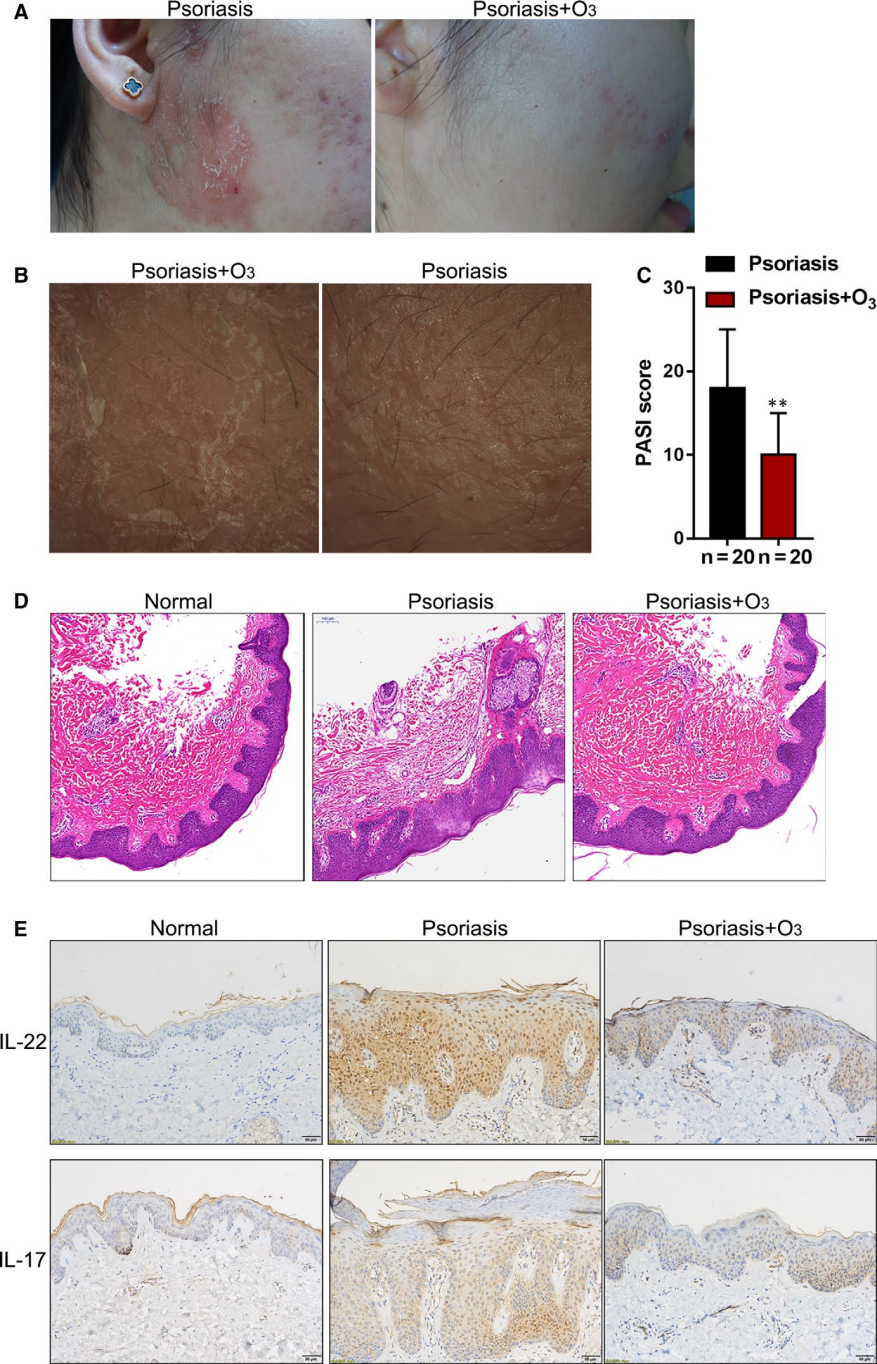
Clinical ozone (O_3_) therapy improves psoriatic lesions in vivo. A and B, The appearance of the lesions in patients before and after receiving the ozone therapy. C, PASI scores of 20 patients before and after ozone therapy. D, Histopathological characteristics of normal skin (NC, negative control), psoriasis lesion without the ozone therapy and psoriasis lesion with the ozone therapy were shown by H&E staining. E, IL‐22 and IL‐17 levels in normal skin, psoriasis lesion without the ozone therapy and psoriasis lesion with the ozone therapy were shown by IHC staining

The appearance of the lesions in patients before and after receiving the ozone therapy is shown in Figure 1A,B to demonstrate the efficiency of the ozone therapy. Before the ozone treatment, there were obvious erythema, plaque, infiltration and scales on the psoriasis lesion area. After 4 weeks of treatment, the mentioned erythema, plaque, infiltration and scales were all improved, and some pigmentation remained. As further revealed by PASI scores, the PASI scores of patients after ozone therapy were significantly reduced, compared to that before ozone therapy (Figure 1C). Histopathological characteristics of normal skin (NC, negative control), psoriasis lesion without the ozone therapy and psoriasis lesion with the ozone therapy were shown by H&E staining. Consistent with the appearance, there were acanthosis, hyperkeratosis with parakeratosis, dermal vasodilation and extracellular extravasation in the psoriasis lesions, and the ozone therapy effectively reversed the appearance and pathological changes in psoriatic lesions (Figure 1D). The cytokines IL‐17 and IL‐22 increased in psoriatic lesion tissues while decreased under ozone therapy (Figure 1E).

### 3.2 The ozone therapy modulates the protein levels of KRT6/10 in psoriasis lesions in vivo

**Figure 2:**
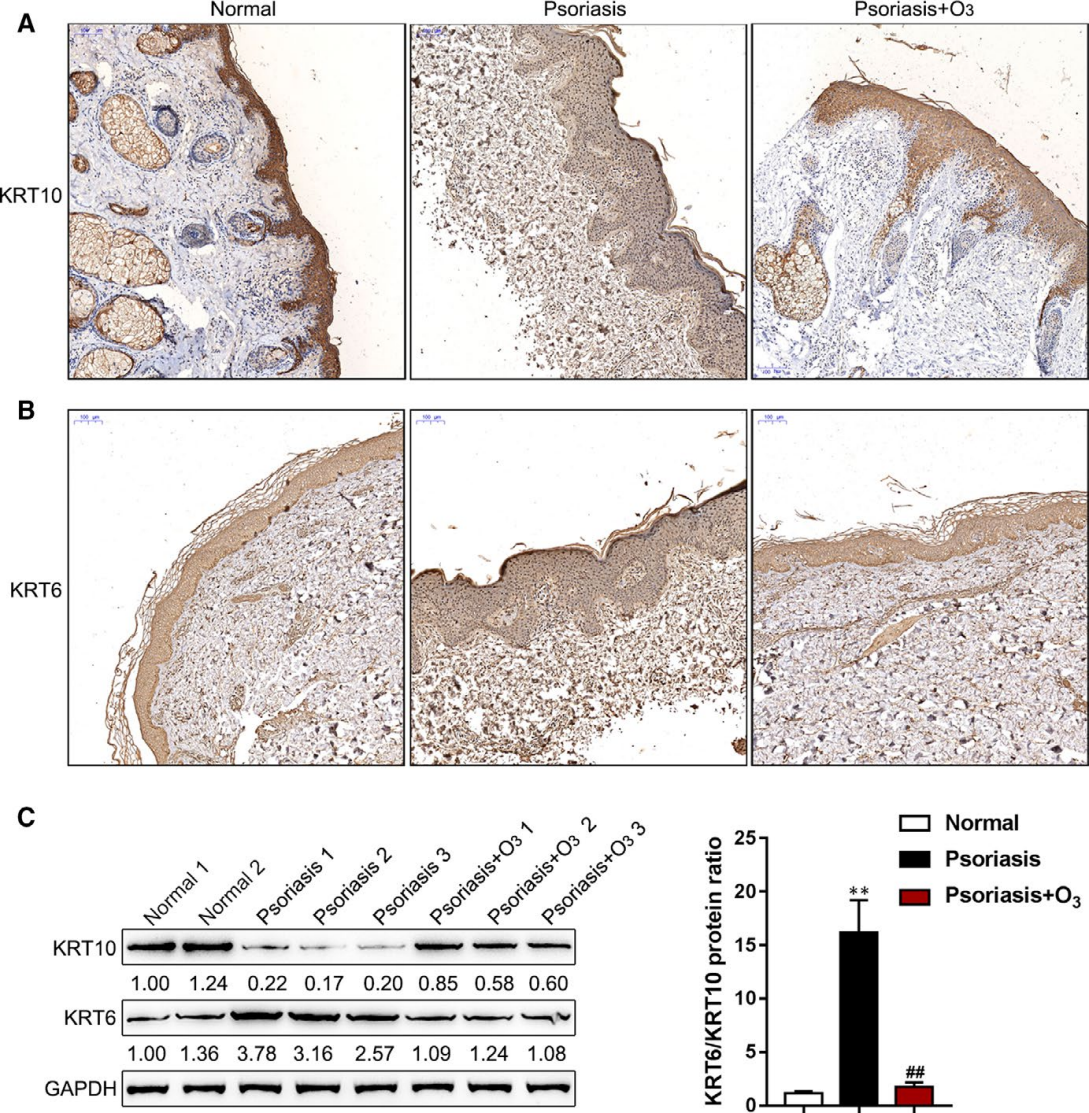
The ozone therapy modulates the protein levels of KRT6/10 in psoriasis lesions in vivo. A and B, The levels of KRT6 and KRT10 in normal skin, psoriasis lesions with or without the ozone therapy were examined by immunohistochemical (IHC) staining. C, The protein levels of KRT6 and KRT10 were examined in psoriasis lesions with or without the ozone therapy by immunoblotting. The ratios of KRT6/KRT10 in three groups were shown

As we have mentioned, the reduction of KRT10 and other differentiation markers is one of the characteristics of psoriasis.^27^ To investigate whether the ozone therapy improves psoriasis through KRT6/10, the levels of KRT6 and KRT10 in normal skin, psoriasis lesions with or without the ozone therapy were examined by immunohistochemical (IHC) staining. As shown in Figure 2A,B, ozone treatment increased the content of KRT10 while decreased KRT6, suggesting that ozone treatment promotes the differentiation and maturity of basal keratinocytes. Immunoblotting further confirmed that ozone treatment significantly increased the protein levels of KRT10 while decreased KRT6 expression in psoriasis lesions (Figure 2C); moreover, the ratio of KRT6/KRT10 was significantly increased in psoriasis lesion while reduced after ozone therapy (Figure 2C), indicating that KRT6/10 might be involved in the effects of ozone on psoriasis lesions.

### 3.3 The ozone therapy improves IMQ‐induced psoriasis‐like dermatitis in vivo

**Figure 3:**
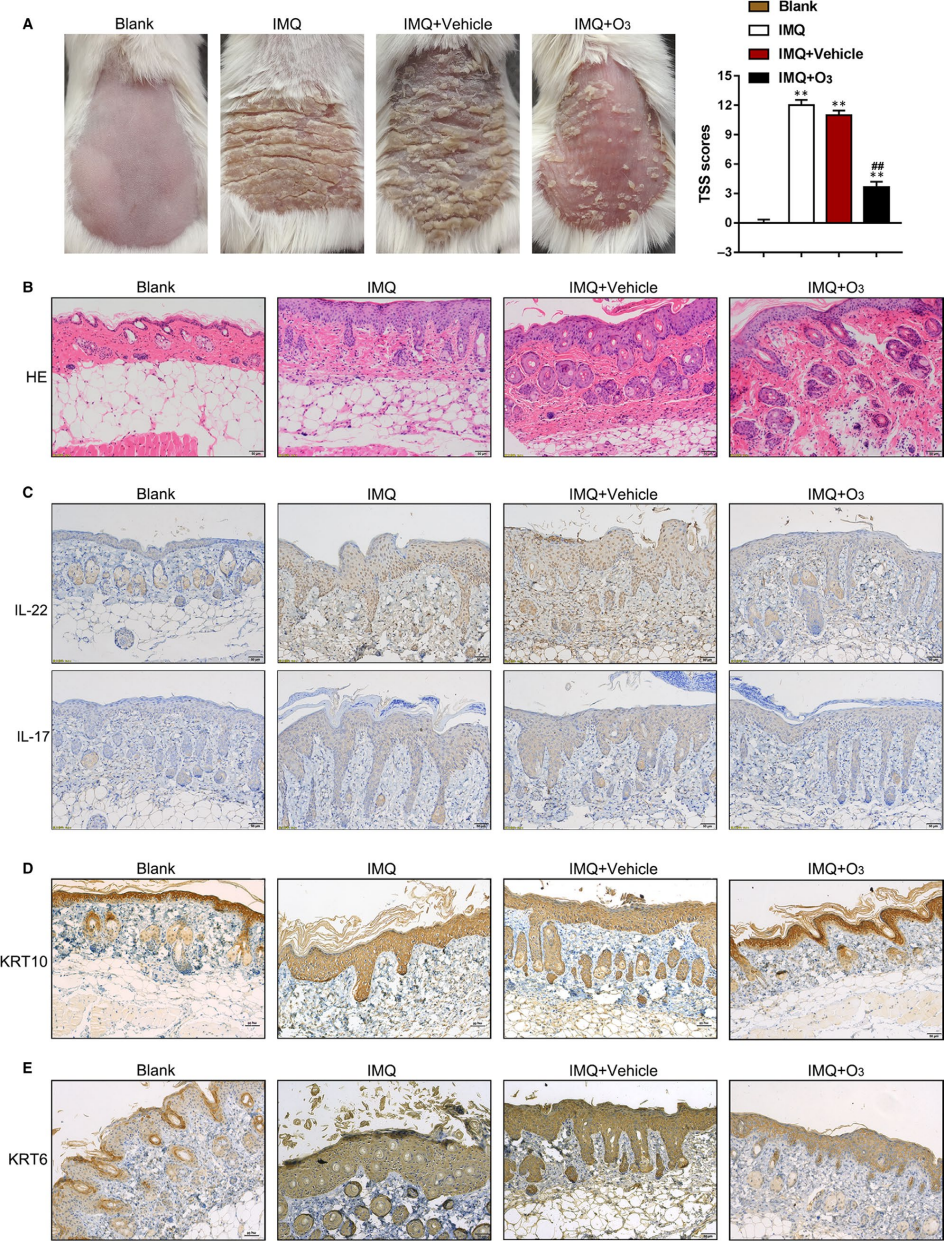
The ozone therapy improves IMQ‐induced psoriasis‐like dermatitis in vivo. A, IMQ‐induced psoriasis‐like dermatitis model was established in mice. The appearance of the dorsal skin in blank, IMQ, IMQ + Vehicle and IMQ + Ozone groups was shown. The TSS scores were evaluated. B, Pathological changes were examined by HE staining. C‐E, The protein contents of IL‐22 (C the up panel), IL‐17 (C the low panel), KRT6 (D) and KRT10 (E) in skin tissues in four groups examined by IHC staining

To further confirm that KRT6 and KRT10 are involved in the effects of the ozone therapy on psoriasis, we established psoriasis‐like dermatitis model in mice by applying IMQ cream. Mice were subjected to blank, IMQ, IMQ + Vehicle, or IMQ + ozone treatment and examined for the appearance of the dorsal skin; the dorsal skin of mice from IMQ and IMQ + Vehicle groups presented as a typical psoriasis‐like dermatitis, whereas the ozone therapy remarkably improved the psoriasis‐like appearance (Figure 3A). Consistently, the TSS scores in IMQ and IMQ + Vehicle groups remarkably increased whereas the ozone therapy significantly reduced the TSS scores (Figure 3A). H&E staining showed the typical histopathological features of psoriasis‐like dermatitis including parakeratosis and Munro's micro‐abscesses, obvious acanthosis with rete ridges extended and large inflammatory cells infiltrated in both IMQ and IMQ + Vehicle groups; the ozone therapy partially reversed these pathological changes (Figure 3B). IHC staining showed that psoriasis‐related cytokines IL‐17 and IL‐22 were increased in IMQ and IMQ + Vehicle groups compared to those in control group while ozone therapy decreases their expression (Figure 3C). In Figure 3D,E, the numbers of KRT6‐positive cells were prominently higher, whereas the numbers of KRT10‐positive cells were lower in IMQ and IMQ + Vehicle groups compared to those in control group (Figure 3D,E); the ozone therapy increased KRT10, whereas decreased KRT6 protein (Figure 3D,E). The above data indicate the successful establishment of IMQ‐induced psoriasis‐like dermatitis model in mice, and the ozone therapy alters the contents of KRT6 and KRT10 proteins.

### 3.4 Ozone promotes the differentiation of keratinocytes in vitro

**Figure 4:**
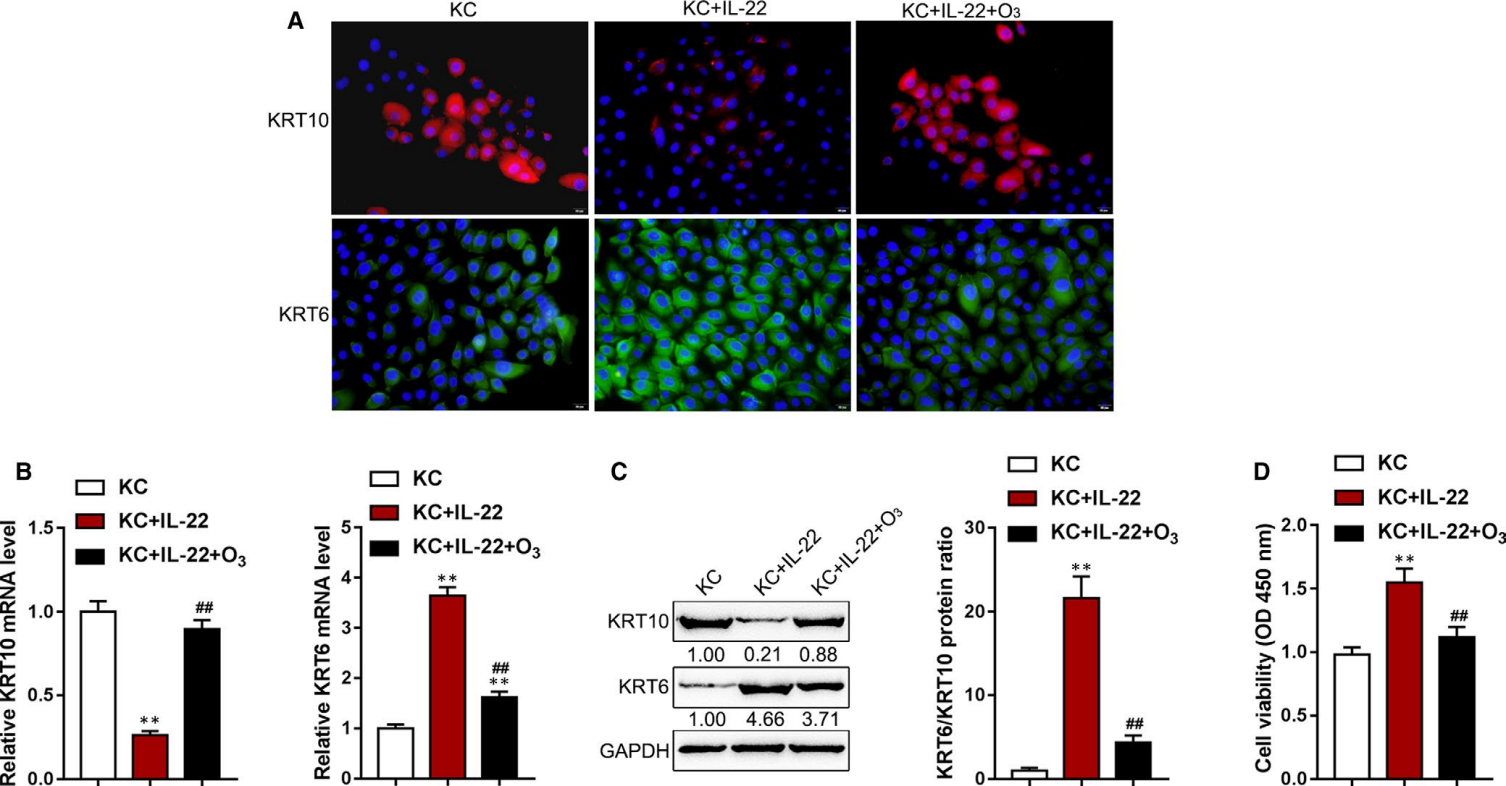
Ozone promotes the differentiation of keratinocytes in vitro KCs were stimulated with IL‐22 in the presence or absence of ozone therapy and examined for (A) the content and distribution of KRT6 and KRT10 in KCs in response to the ozone treatment by IF staining; (B) the mRNA expression of KRT6 and KRT10 by real‐time PCR; (C) the protein levels of KRT6 and KRT10 by immunoblotting; the ratio of KRT6/KRT10 was shown; and (D) the cell viability of KCs by MTT assays. **P < .01

To further investigate the molecular mechanism of ozone improving psoriasis, we evaluated the cellular effects of ozone on primary KCs. We established a cell model of psoriasis by stimulating KCs with IL‐22 and then treated the KCs with the ozone therapy. Next, we examined the content and distribution of KRT6 and KRT10 in primary KCs in response to the ozone stimulation by IF staining. As shown in Figure 4A, the intensity of the fluorescence representing KRT10 was attenuated by IL‐22 stimulation whereas enhanced by ozone treatment; the intensity of the fluorescence representing KRT6 enhanced by IL‐22 stimulation whereas weakened by the ozone therapy. As a further confirmation, real‐time PCR analyses revealed that KRT10 mRNA expression could be remarkably inhibited by IL‐22 but rescued by ozone treatment; in contrast, KRT6 mRNA expression was significantly promoted by IL‐22 but inhibited by ozone treatment (Figure 4B). Similar results were revealed by Immunoblotting (Figure 4C). Moreover, since KRT6 is considered as an activation marker for KCs,^43^, ^44^ next, we investigated the cell viability of KCs in response to ozone therapy. As shown in Figure 4D, IL‐22 stimulation significantly promoted, whereas ozone treatment significantly suppressed the proliferation of KCs, suggesting that ozone treatment might inhibit KRT6 to suppress the excessive proliferation of KCs. These data indicate that the ozone therapy improves psoriasis through modulating the differentiation of basal keratinocytes.

### 3.5 Tp63 is involved in ozone‐mediated KC differentiation in vitro

**Figure 5:**
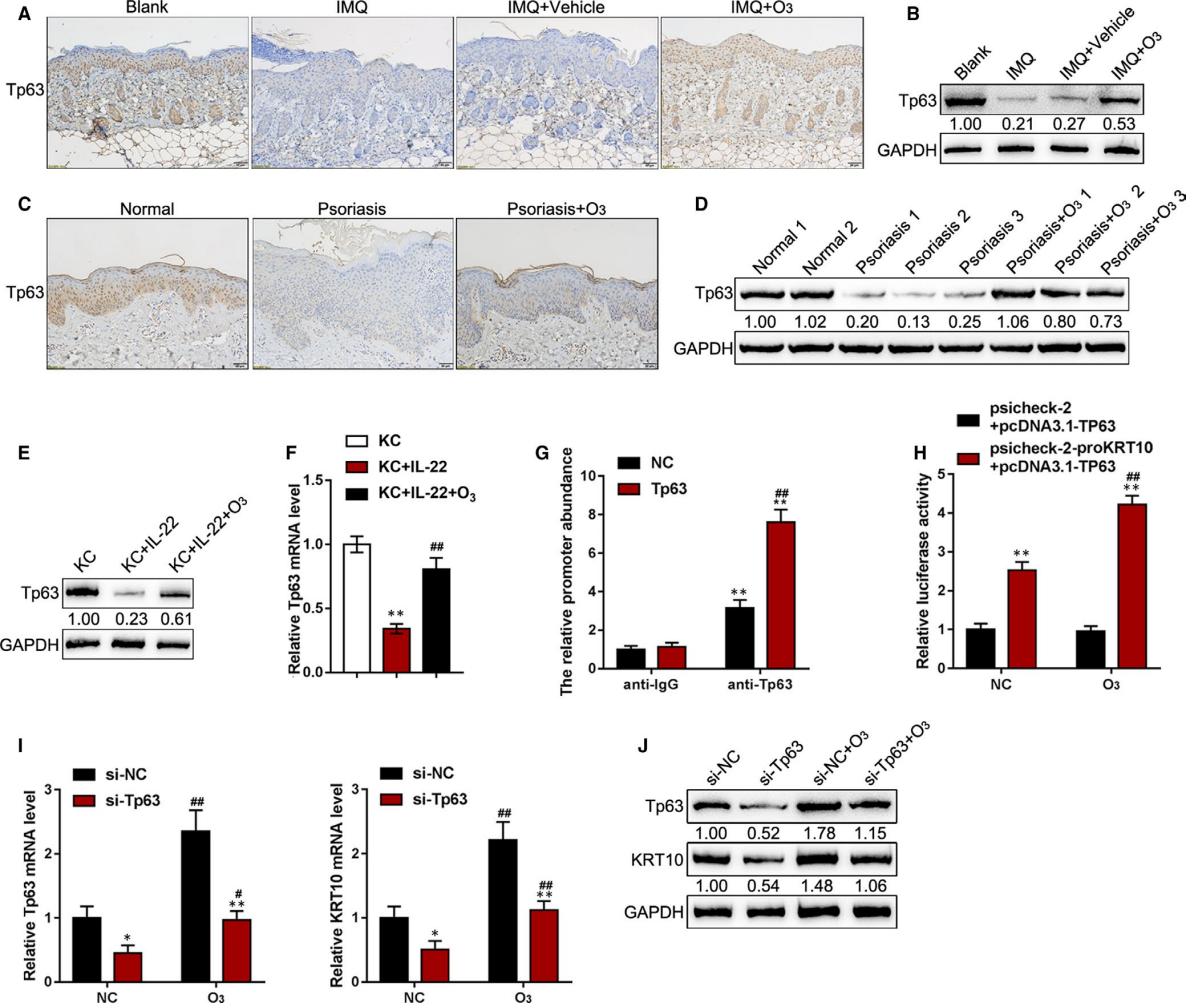
Tp63 is involved in ozone‐mediated KC cell differentiation in vitro. A and B, the protein levels of Tp63 were examined in blank, IMQ, IMQ + Vehicle and IMQ + Ozone groups by IHC staining (A) and immunoblotting (B). C and D, The protein levels of Tp63 were examined in normal skin samples and psoriasis lesions with or without the ozone therapy by IHC staining (C) and immunoblotting (D). E and F, KCs were stimulated with IL‐22 and examined for the mRNA expression (F) and protein levels of Tp63 (E) with or without ozone treatment. G, ChIP assays were performed in 293T cells with anti‐Tp63 antibody to validate the binding of Tp63 to KRT10 promoter region. H, Luciferase reporter assays were performed in 293T cells to validate the effects of Tp63 on KRT10 transcription activity. I and J, KCs were transfected with si‐Tp63 in the presence or absence of ozone treatment and examined for the mRNA expression and protein levels of Tp63 and KRT10 by real‐time PCR (I) and immunoblotting (J). *P < .05, **P < .01, compared to the control group; ^#^ *P* < .05, ^##^ *P* < .01, compared to the non‐treated group

Tp63 was found to bind to the KRT10 promoter region in keratinocytes by the Chip‐Atlas (http://chip-atlas.org/) Chromatin Immunoprecipitation Database (http://ddbj.nig.ac.jp/). To investigate whether Tp63 is involved in keratinocyte differentiation in psoriasis, we next examined the protein levels of Tp63 in IMQ‐induced psoriasis‐like dermatitis mice model and in human normal skin tissues and psoriasis lesions with or without the ozone therapy by IHC staining and Immunoblotting. As shown in Figure 5A,B, the protein levels of Tp63 were significantly decreased in IMQ and IMQ + Vehicle groups compared to control group while ozone therapy increased Tp63 expression. As shown in Figure 5C,D, the protein levels of Tp63 were remarkably down‐regulated in lesion tissues without ozone therapy, compared to those in normal skin tissues; ozone treatment increased the protein levels of Tp63 in psoriasis lesions. To investigate the molecular mechanism, we treated KCs with or without ozone and then examined Tp63 mRNA and protein expression. As shown in Figure 5E,F, IL‐22 treatment remarkably reduced, whereas ozone treatment remarkably enhanced Tp63 mRNA and protein expression within KCs. Next, ChIP assays were performed using anti‐Tp63 to validate the predicted binding of Tp63 to KRT10 promoter region. As shown in Figure 5G, the levels of promoter abundance binding to Tp63 antibody were significantly higher, compared to those binding to IgG. We validated the effects of Tp63 on KRT10 transcription activity via performing luciferase reporter assays. As shown in Figure 5H, the luciferase activity of KRT10 promoter vector (psiCheck‐2‐proKRT10) could be significantly promoted by co‐transfection with pcDNA3.1/TP63 and further enhanced by ozone treatment, indicating that ozone treatment induces Tp63 expression, therefore promoting the transcription of KRT10.

To further confirm the above findings, KCs were transfected with si‐Tp63 with or without ozone treatment and examined for mRNA and protein expression in Tp63 and KRT10. Tp63 silence significantly decreased, while ozone treatment significantly increased mRNA and protein expression in Tp63 and KRT10; Tp63 silence remarkably attenuated the promotive effects of ozone on Tp63 and KRT10 (Figure 5I,J). In summary, Tp63 can activate the transcription of KRT10 via targeting its promoter region; ozone promotes the differentiation of basal keratinocytes via Tp63/KRT10.

### 3.6 Expression and correlation of Tp63 and KRT10 in tissue samples in vivo

**Figure 6:**
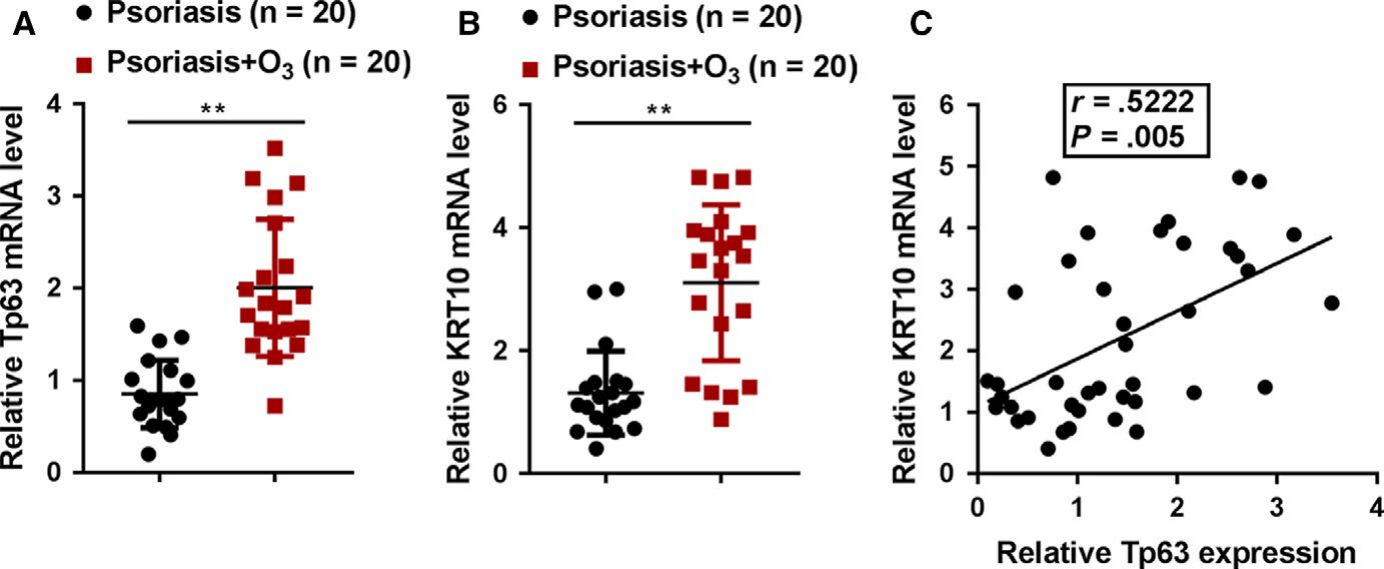
Expression and correlation of Tp63 and KRT10 in tissue samples in vivo. A and B, The expression of Tp63 and KRT10 in psoriasis lesion tissues with or without ozone treatment were determined by real‐time PCR. C, The correlation of Tp63 and KRT10 expression in tissue samples was analysed by Pearson's correlation analysis

To provide further evidence, we examined Tp63 and KRT10 expression in psoriasis lesion tissues with or without ozone treatment. As shown in Figure 6A,B, Tp63 and KRT10 mRNA expression could be remarkably up‐regulated upon ozone treatment in psoriasis lesions, compared to those in non‐treated psoriasis lesions. The expression of Tp63 and KRT10 was positively correlated in tissue samples as revealed by Pearson's correlation analysis (Figure 6C).

## 4 DISCUSSION

In the present study, representative images showing the treatment efficiency of the ozone therapy on psoriasis were shown. KRT6, IL‐17 and IL‐22 protein within psoriasis lesions was significantly decreased, while KRT10 protein in psoriasis lesions was increased by ozone treatment. In the meantime, IL‐22 induced psoriatic changes in KCs, and ozone treatment significantly down‐regulated KRT6 mRNA and protein expression while up‐regulated KRT10 mRNA and protein expression within primary KCs. Moreover, Tp63 bound to KRT10 promoter region to activate its transcription in basal keratinocytes; the promotive effects of ozone on Tp63 and KRT10 were significantly reversed by TP63 silence. Both Tp63 and KRT10 mRNA expression were significantly increased by ozone treatment in psoriasis lesions; there was a positive correlation between Tp63 and KRT10 mRNA expression within tissue samples, suggesting that ozone induces the expression of Tp63 to enhance the expression of KRT10 and the differentiation of basal keratinocytes, therefore improving the psoriasis.

The application of ozone therapy in psoriasis has been reported previously. However, oxygen‐ozone (O_2_/O_3_) therapy administered by autohemotransfusion has been reported to cause the gas embolism, which could directly lead to unexpected death of the patient.^45^ In the present study, ozone was dissolved in plant oil and applied to the lesion skin to avoid decomposition of ozone into oxygen at room temperature and the gas embolism. The morphometric and pathological examinations revealed that ozonated oil application improved the psoriasis; the red spots, plaques, infiltration and scales are basically subsided after treatment, further indicating that the ozone therapy is an effective and safe strategy for psoriasis.

Psoriasis is a commonly seen, chronic and inflammatory dermatosis, which is characterized by immune cell infiltration in the dermis and epidermis and excessive proliferation and abnormal differentiation of keratinocytes.^46^ As Th17 and Th22 secreted cytokines, IL‐17 and IL‐22 induce keratinocyte immune activation and hyperplasia.^47^ In our present study, as expectedly, both IL‐17 and IL‐22 levels were increased in psoriasis lesions in patients and IMQ mouse models. The ozone therapy effectively reduced the up‐regulation of IL‐17 and IL‐22 in psoriasis lesions. The researches on epidermal hyperproliferation and abnormal differentiation in psoriasis have demonstrated the presence of hyperproliferation‐related KRTs. As the type I keratin chaperone of K6, KRT16 is involved in intermediated filament heterodimer formation.^48^ This KRT6/ KRT16 keratin pair is not found within normal epidermis, but is induced within hyperplasia cells, including psoriatic epidermis.^49^, ^50^ Nevertheless, at the beginning of final differentiation, the keratinocytes first move up to become mitotic cells and then convert to keratin pair KRT1‐KRT10 expression.^51^ Within hyperplasia conditions, such as psoriasis, down‐regulation of KRT10 occurs in keratinocytes. It is known that, in traditional treatment for psoriasis, topical steroids can be used to promote normal differentiation and reduce hyperplasis in the course of psoriatic plaque treatment. These alterations can be manifested as increased KRT10 and decreased KRT6, respectively.^52^ Consistent with previous studies, we observed up‐regulated KRT6 and down‐regulated KRT10 protein levels in psoriasis lesions of patients and IMQ mouse models; in the meantime, ozone treatment significantly reduced the KRT6 while increased the KRT10 levels. Further in vitro results also showed that IL‐22 stimulation significantly increased KRT6 and inhibited KRT10 expression, whereas ozone treatment on primary KCs significantly decreased KRT6 mRNA expression and protein levels while increased KRT10 mRNA expression and protein levels. More importantly, the cell viability of KCs could be significantly suppressed by ozone treatment, indicating that the ozone therapy might improve psoriasis through suppressing the excessive proliferation while promoting the differentiation of the basal keratinocytes.

As we have mentioned, KRT10 expression is significantly down‐regulated in psoriasis lesions while rescued by ozone treatment. To further understand the mechanism of KRT10 deregulation in the pathogenesis of psoriasis, we analysed online data in the chromatin co‐immunoprecipitation database (http://ddbj.nig.ac.jp/) via the Chip‐Atlas (http://chip-atlas.org/) and found that Tp63 could activate the transcription of KRT10 via targeting its promoter region. TAp63 isoforms have been considered to provide a contribution to epidermal differentiation.^53^ In the process of keratinocyte differentiation of p53‐mutant HaCaT cells, TAp63α can be related to the ability of growth differentiation factor 15 (GDF15) to up‐regulate and to secrete. TAp63 activates the transcription of GDF15 via targeting its promoter region; the down‐regulation of GDF15 could promote cell proliferation and reduce the expression of KRT10 and other differentiation markers upon the stimulation of differentiation.^54^ Herein, the predicted binding of Tp63 to KRT10 promoter region was validated. Ozone treatment significantly induced TP63 mRNA expression and protein levels, therefore increasing KRT10 protein levels. After TP63 silence, ozone‐induced increases in Tp63 and KRT10 protein levels were significantly reversed. These data indicate that ozone treatment promotes the differentiation of basal keratinocytes via Tp63/KRT10, finally improving psoriasis.

As a further confirmation, Tp63 mRNA and KRT10 mRNA expression were significantly up‐regulated in ozone‐treated psoriasis lesions; Tp63 and KRT10 mRNA expression in tissue samples was positively correlated. In conclusion, the application of ozonated oil could be an efficient and safe treatment for psoriasis; ozone promotes the differentiation of basal keratinocytes via increasing Tp63‐mediated transcription of KRT10, therefore improving psoriasis.

## CONFLICT OF INTEREST

The authors declare that they have no competing interests.

## AUTHOR CONTRIBUTIONS

Lihua Gao, Jianhua Dou, Ziqiang Luo and Jianyun Lu contributed to experimental design and supervising the whole experimental process; Aiyuan Guo, Haipeng Cheng and Caifeng Yang were involved in the experimental conducting; Bo Zhang, Jinrong Zeng, Qingmei Cheng, Li Lei, Lina Tan, Qinghai Zeng and Shu Ding contributed to the data analysis and manuscript preparation. All the authors read, revised and approved the final manuscript.

## ETHICAL APPROVAL AND CONSENT TO PARTICIPATE

All procedures performed in studies involving human participants and animals were in accordance with the ethical standards of The Third Xiangya Hospital and with the 1964 Helsinki declaration. Informed consent to participate in the study has been obtained from participants.

## Data Availability

Please contact the authors for data requests.
